# Magnesium Oxide-Modified Alumina for Enhanced Adsorption of Multi-Sulfonated Azo Dyes: Performance and Mechanistic Insights

**DOI:** 10.3390/molecules31132364

**Published:** 2026-07-05

**Authors:** Boning Jiang, Shuaiqi Chen, Xuhui Wang, Yujian Sun, Yaowen Wang, Wei Chu, Shuaijie Tang, Junwei Jia, Xiuchao Fu, Yue Bai, Xiangyu Xu, Jiaqing Song

**Affiliations:** 1 College of Chemistry, Beijing University of Chemical Technology, Beijing 100029, China; 2023400285@mail.buct.edu.cn (B.J.); 2022430033@mail.buct.edu.cn (S.C.); 2024201022@mail.buct.edu.cn (Y.W.); chuwei8991@163.com (W.C.); 2025210857@mail.buct.edu.cn (S.T.); 2025200932@mail.buct.edu.cn (J.J.); 2025210780@mail.buct.edu.cn (X.F.); 2025210824@mail.buct.edu.cn (Y.B.);; 2 State Key Laboratory of Chemical Resource Engineering, Beijing University of Chemical Technology, Beijing 100029, China; 3 Sinopec Catalyst Co., Ltd., Beijing 100176, China; xuhuiwang@126.com; 4 Graduate School, Anshan Normal University, Anshan 114007, China; sunyujian50295@163.com

**Keywords:** MgO-modified alumina, sunset yellow, amaranth, adsorption, ion exchange, surface hydroxyl groups

## Abstract

Multi-sulfonated anionic azo dyes are difficult to remove from water because their high solubility and ionized sulfonate groups reduce their affinity toward many oxide adsorbents. In this study, magnesium oxide-modified alumina composites were prepared by loading magnesium oxide onto mesoporous alumina to increase the density of surface hydroxyl groups. Sunset Yellow and Amaranth were selected as model dyes containing two and three sulfonate groups, respectively. Compared with pristine alumina, the modified adsorbents exhibited higher isoelectric points, stronger hydroxyl-related infrared signals, and significantly enhanced adsorption capacities. At pH 4.0, the maximum adsorption capacities reached 787 mg/g for Sunset Yellow and 709 mg/g for Amaranth. Notably, MgA-2 exhibited the highest utilization efficiency of MgO active sites. Adsorption kinetics were well described by the pseudo-second-order model, while equilibrium data followed the Langmuir model. Mechanistic analysis indicated that adsorption process mainly proceeded through ion exchange between surface hydroxyl groups and dye sulfonate groups. The higher adsorption capacity of Sunset Yellow was attributed to its lower number of sulfonate groups and lower demand for hydroxyl binding sites. These results demonstrate that magnesium oxide modification is an effective strategy for enhancing alumina-based adsorbents for the removal of multi-sulfonated anionic dyes from water.

## 1. Introduction

Anionic azo dyes are widely used in the food, textile, printing, pharmaceutical, and cosmetic industries due to their strong coloration, high aqueous solubility, and excellent chemical stability [1,2]. However, these same properties also contribute to their persistence in aquatic environments once discharged into wastewater streams. Untreated dye-containing effluents can increase water coloration, reduce light penetration, inhibit photosynthetic activity, and pose potential toxicological risks associated with certain azo dye structures, including mutagenic and carcinogenic effects [3,4,5]. Therefore, the efficient removal of dyes from industrial wastewater is of considerable environmental importance. Various treatment technologies, including adsorption [4], membrane separation [6], advanced oxidation [7], electrochemical treatment [8], and biological treatment [9], have been developed for dye-containing wastewater. Among these methods, adsorption is considered one of the most effective approaches because of its high removal efficiency, operational simplicity, and low cost [10,11]. A wide range of adsorbents, such as activated carbon [12], alumina [13], metal–organic frameworks (MOFs) [14], and chitosan [15,16], have been employed for dye removal from wastewater. Among them, alumina (Al_2_O_3_) is regarded as a promising adsorbent for water-treatment applications due to its low cost, environmental friendliness, high chemical stability, adjustable surface properties, and ease of scale-up production [17,18,19].

In our previous studies, phosphorus modification partially replaced the surface Al–OH groups of alumina with Al–O–P bonds, thereby suppressing interparticle sintering during high-temperature dehydroxylation and enabling the preparation of alumina with both high specific surface area and large pore volume [20,21]. However, despite these favorable pore structure characteristics, the number of surface active sites remained relatively limited, which restricted the adsorption and separation performance of the material [22,23]. Wang et al. [24] found that loading Mg onto mesoporous alumina increased the total basicity from 0.224 to 0.773 mmol/g and enhanced the CO_2_ adsorption capacity from 0.50 to 1.08 mmol/g. Liu et al. [25] reported that MOF@γ-Al_2_O_3_ materials exhibited significantly higher adsorption capacities for anionic dyes, with the adsorption capacity for Congo red reaching 1390–1540 mg/g. He et al. [26] demonstrated that La/Ce-modified mesoporous alumina showed improved adsorption performance, with a maximum adsorption capacity for F^−^ of 26.5 mg/g and an increased isoelectric point (IEP) of 10.2. These studies suggest that the introduction of additional components can increase the number of surface active sites and alter the surface properties of Al_2_O_3_, thereby enhancing its adsorption performance. Among various modifiers, magnesium oxide (MgO) has attracted considerable attention because of its strong surface basicity, abundant surface hydroxyl groups, and high affinity toward acidic or anionic species [27,28]. When dispersed on porous alumina, MgO can introduce additional hydroxyl-rich adsorption sites while maintaining the favorable pore structure of Al_2_O_3_. Such modification is expected to increase the positively charged surface region under acidic to near-neutral conditions and promote interactions with anionic dye molecules. Moreover, the aggregation of MgO particles may limit the effective utilization of active sites, whereas supporting MgO on porous Al_2_O_3_ can improve its dispersion and enhance the accessibility of adsorption active sites [29]. Therefore, MgO-modified Al_2_O_3_ is considered a promising adsorbent for the efficient removal of anionic dyes.

**Figure 1:**
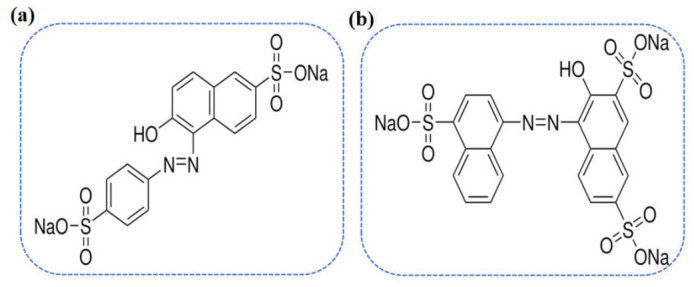
Molecular structure of (**a**) SY and (**b**) AM.

Sunset Yellow (SY) [30] and Amaranth (AM) [31] are representative water-soluble anionic azo dyes containing multiple sulfonate groups. As shown in Figure 1, each SY molecule contains two sulfonate groups, whereas each AM molecule contains three sulfonate groups. These sulfonate groups not only enhance the water solubility and hydrophilicity of the dye molecules but also play a crucial role in determining their interactions with positively charged or hydroxyl-rich adsorbent surfaces. Therefore, SY and AM are suitable model compounds for investigating the influence of sulfonate group number on adsorption behavior over hydroxyl-rich oxide surfaces. Understanding this relationship is important for elucidating the adsorption mechanism of multi-sulfonated dyes and for guiding the rational design of oxide-based adsorbents.

In this work, MgO-modified Al_2_O_3_ composites were prepared by loading MgO onto mesoporous Al_2_O_3_, and their adsorption performance toward SY and AM was systematically investigated. The structure and surface properties of the adsorbents were characterized by X-Ray diffraction, elemental mapping, N_2_ adsorption–desorption, isoelectric point analysis, and Fourier-transform infrared spectroscopy. The effects of MgO loading, contact time, initial dye concentration, adsorbent selectivity, and solution pH on adsorption performance were examined. Adsorption kinetics and isotherm models were applied to evaluate the adsorption process, while elemental analysis and comparisons of molar adsorption capacities were employed to elucidate the adsorption mechanism. Particular attention was given to the role of surface hydroxyl groups and their interactions with dye sulfonate groups. This work aims to clarify how MgO modification enhances the adsorption capacity of Al_2_O_3_ and to provide mechanistic insight into the adsorption of multi-sulfonated anionic azo dyes on hydroxyl-rich oxide surfaces.

## 2. Results and Discussion

### 2.1. Characterization of Adsorbents

**Figure 2:**
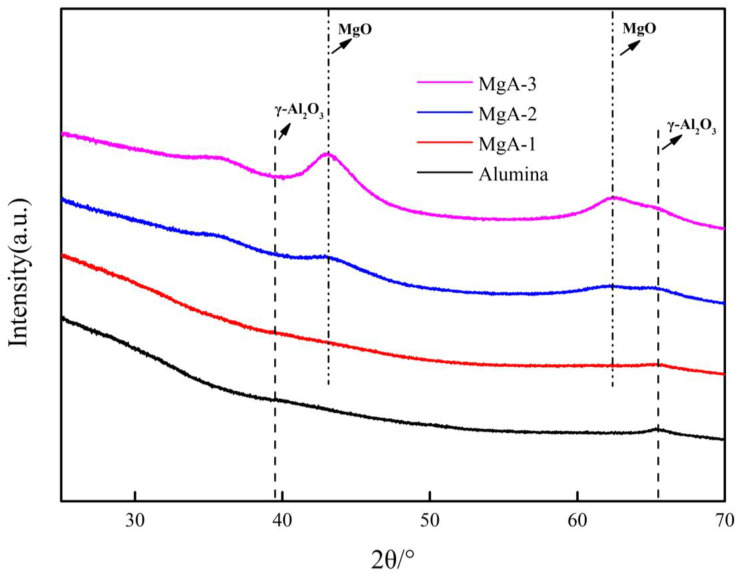
XRD patterns of samples (phosphorus-modified Al_2_O_3_; MgA-x: MgO/Al_2_O_3_ mass ratios of 0.24, 0.48, and 0.72, respectively, x = 1, 2, 3).

The structural and surface properties of the prepared alumina and MgO@Al_2_O_3_ composites (MgA-x) were first evaluated to confirm the successful incorporation of MgO and the evolution of surface active sites. As shown in Figure 2, Alumina, MgA-1, MgA-2, and MgA-3 exhibited characteristic diffraction peaks of γ-Al_2_O_3_ at 2θ = 39.4° and 67.2°, corresponding to the (222) and (440) crystal planes (JCPDS No. 10-0425) [32]. For MgA-1, no distinct diffraction peaks of MgO were observed, indicating that MgO was highly dispersed on the alumina surface. In contrast, MgA-2 and MgA-3 displayed additional diffraction peaks at 2θ = 42.9° and 62.3°, which were assigned to the (200) and (220) planes of MgO (JCPDS No. 45-0946) [32]. With increasing MgO loading, the diffraction peaks of MgO became more intense and sharper, indicating the formation and growth of crystalline MgO on the alumina surface.

**Figure 3:**
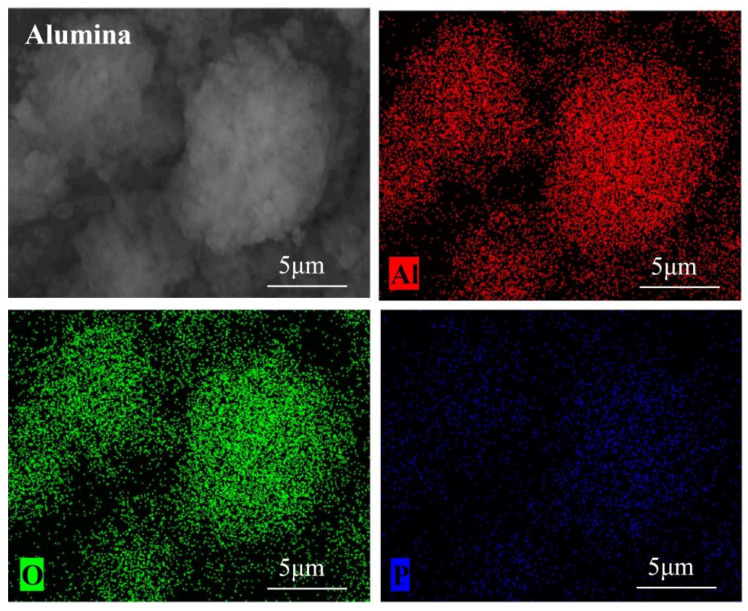
Elemental mapping images of Alumina.

**Figure 4:**
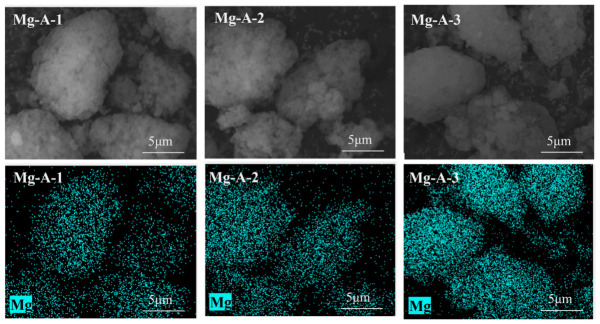
Mg elemental mapping images of MgA-1, MgA-2, and MgA-3.

As shown in Figure 3, elemental mapping revealed that Al, O, and P were uniformly distributed throughout the Alumina substrate, confirming the successful incorporation of phosphorus during synthesis. As shown in Figure 4, the Mg signal increased progressively from MgA-1 to MgA-3 with increasing MgO loading. Mg species were relatively well dispersed in MgA-1 and MgA-2, whereas localized Mg-rich regions became more evident in MgA-3. This loading-dependent dispersion behavior was expected to influence the exposure and accessibility of MgO-related surface active sites.

**Figure 5:**
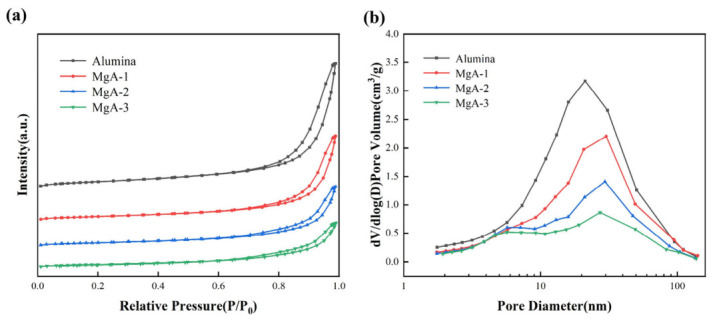
(**a**) N_2_ adsorption–desorption isotherms and (**b**) pore size distributions of the samples.

As shown in Figure 5a, the N_2_ adsorption–desorption isotherms of all samples exhibited type IV behavior, indicating a mesoporous structure. Figure 5b showed that the pore size distribution of Alumina and MgA-1 was unimodal, centered at approximately 20 nm, whereas MgA-2 and MgA-3 displayed bimodal distributions, likely due to additional interparticle pores formed after MgO deposition. As summarized in Table 1, BET analysis revealed that the specific surface area decreased from 548 m^2^/g for Alumina to 254 m^2^/g for MgA-3, while the pore volume decreased from 2.97 to 1.11 cm^3^/g. The reduction in surface area and pore volume with increasing MgO loading can be attributed to pore blockage and the coverage of Alumina surface sites, highlighting a trade-off between the total surface area and the number of MgO-modified active sites.

**Figure 6:**
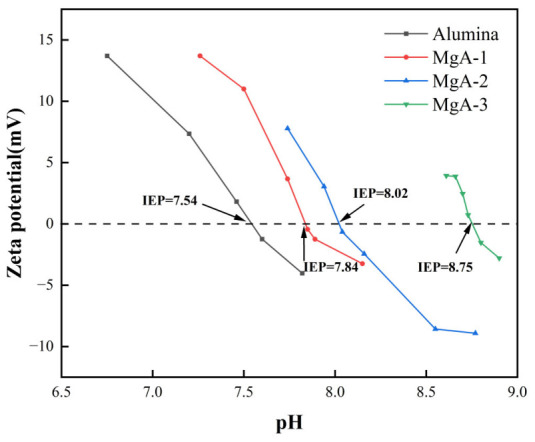
IEP of samples.

As shown in Figure 6, the isoelectric points (IEPs) of the adsorbents increased progressively with MgO loading, from 7.54 for Alumina to 8.75 for MgA-3. A higher IEP is generally associated with a greater number of surface hydroxyl groups that can be protonated [18,33]. The observed increase in IEP indicated that MgA-x maintained positively charged surfaces over a wider pH range. Consequently, the electrostatic attraction toward anionic dyes was enhanced, and ion exchange between surface hydroxyl groups and dye sulfonate groups was promoted [34], thereby improving the adsorption capacities of SY and AM.

**Figure 7:**
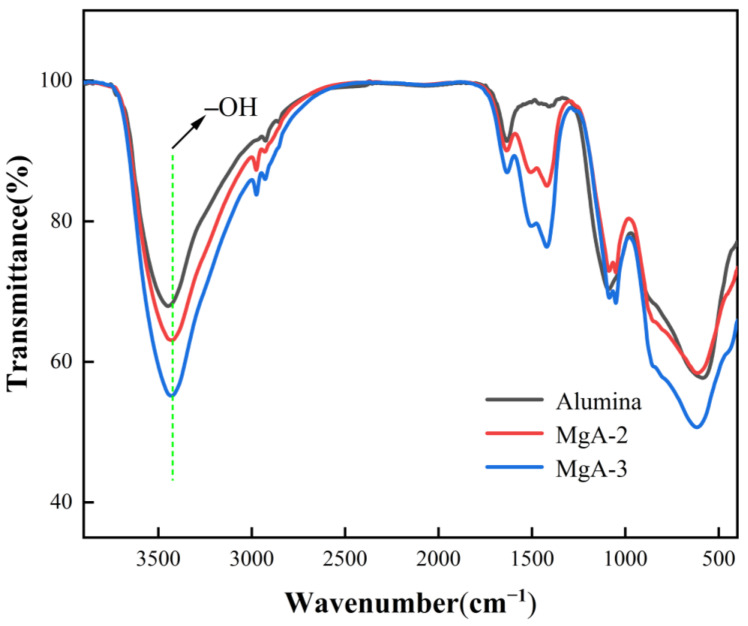
FT-IR spectra of Alumina, MgA-2, and MgA-3.

As shown in Figure 7, Fourier-transform infrared (FT-IR) spectra further supported the enhancement of surface hydroxyl groups after MgO modification. The broad absorption band at 3400–3500 cm^−1^, attributed to the stretching vibrations of –OH groups, became more pronounced in MgA-2 and MgA-3 than in Alumina, indicating that MgO loading increased the abundance of surface hydroxyl active sites. These hydroxyl groups were expected to play a crucial role in ion exchange interactions with the sulfonate groups of anionic dyes.

In summary, the characterization results confirmed the successful incorporation of MgO onto the Alumina surface, resulting in composites with progressively increased Mg content, enriched surface hydroxyl groups, elevated IEP, and preserved mesoporous structures. These modifications established a favorable foundation for enhanced adsorption performance toward multi-sulfonated anionic dyes, as discussed in the following sections.

### 2.2. Adsorption Behavior

#### 2.2.1. Adsorption Kinetics

**Figure 8:**
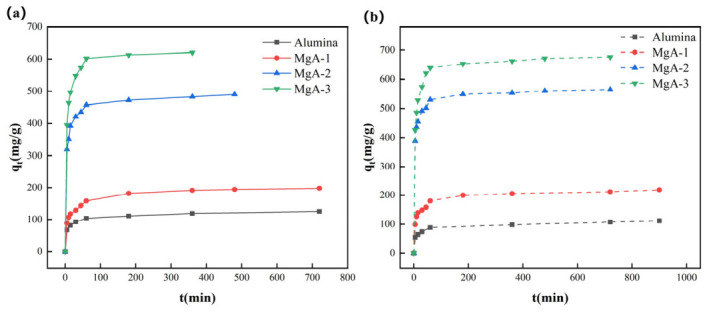
Time-dependent adsorption capacities of the samples toward (**a**) SY and (**b**) AM at pH 4.0.

The adsorption kinetics of SY and AM on Alumina and MgA-x were investigated under an initial dye concentration of 200 mg/L, an adsorbent dosage of 0.1 g/L, and pH 4.0. As shown in Figure 8a,b, all adsorbents exhibited a rapid adsorption stage during the initial period, followed by a slower approach to equilibrium. The equilibrium adsorption capacity increased with MgO loading, reflecting the higher density of accessible surface hydroxyl sites. Notably, owing to its high specific surface area and large pore volume, the prepared Alumina exhibited a much higher adsorption capacity for SY than that reported for γ-Al_2_O_3_ in the literature [35]. For Alumina, equilibrium was reached after 720 min for SY (125.8 mg/g) and 900 min for AM (112.0 mg/g). Although the specific surface area and pore volume decreased after MgO loading, the adsorption performance of MgA-x toward both dyes was still markedly enhanced, indicating that the increase in surface hydroxyl sites played a more critical role in the adsorption process. Among the samples, MgA-3 achieved equilibrium within 360 min for SY (620.2 mg/g) and 720 min for AM (676.1 mg/g), demonstrating that MgO modification not only enhanced the adsorption capacity but also accelerated the adsorption process.

**Figure 9:**
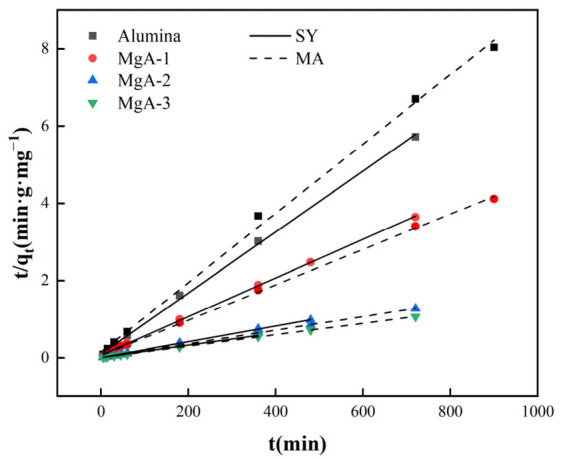
Pseudo-second-order kinetic fitting results for SY and AM adsorption by the samples.

As shown in Figure 9 and Table 2, the kinetic data were fitted using both pseudo-first-order and pseudo-second-order models. For all samples, the pseudo-second-order model exhibited higher determination coefficients (R^2^ > 0.997), and the calculated adsorption capacities were in close agreement with the experimental values. This result indicated that the adsorption of SY and AM was closely related to the adsorbate–adsorbent interactions and the availability of surface active sites.

#### 2.2.2. Adsorption Isotherm Models

**Figure 10:**
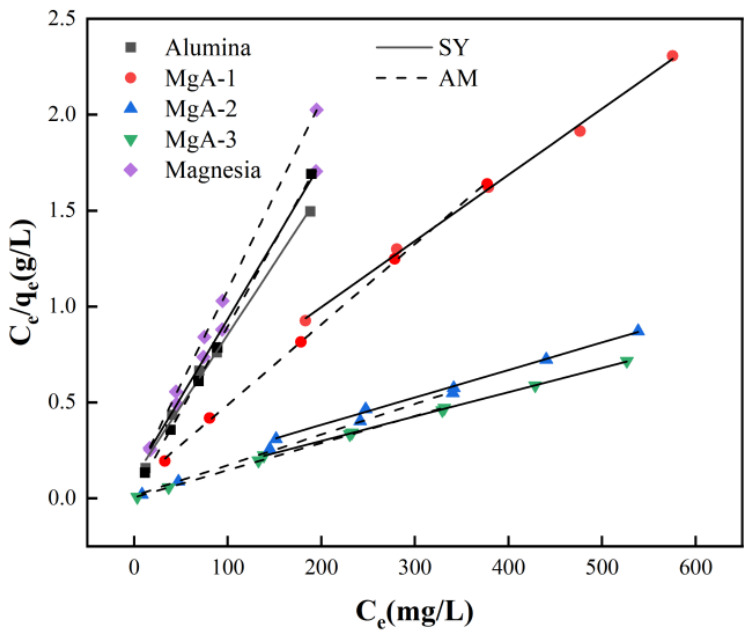
Langmuir fitting curves for SY and AM adsorption by the samples.

The equilibrium adsorption of SY and AM was further investigated by varying the initial dye concentration, and the resulting data were fitted using the Langmuir and Freundlich isotherm models. As shown in Figure 10 and Table 3 and Table 4, the Langmuir model provided a better fit for all samples (R^2^ > 0.994), indicating monolayer adsorption on relatively homogeneous sites. The maximum Langmuir adsorption capacities for Magnesia were 122.4 and 101.3 mg/g for SY and AM, respectively. The maximum Langmuir adsorption capacities increased with MgO loading, from 134.4 mg/g for SY on Alumina to 787.4 mg/g on MgA-3, and from 113.6 mg/g for AM on Alumina to 709.2 mg/g on MgA-3. These results demonstrated that MgO modification effectively increased the number of surface active sites and significantly enhanced the adsorption capacity of the adsorbents.

Although the overall adsorption capacity increased with increasing MgO loading, the adsorption capacity per unit mass of MgO exhibited a different trend. After subtracting the adsorption contribution of the alumina support, the adsorption capacities attributed to MgO in MgA-1, MgA-2, and MgA-3 were calculated to be 937.8, 1552.2, and 909.8 mg/g MgO for SY, and 760.0, 1441.8, 820.7 mg/g MgO for AM, respectively. Among them, MgA-2 exhibited the highest adsorption capacity per unit mass of MgO, indicating that the utilization efficiency of MgO active sites was the highest at this loading level. Notably, the MgO-normalized adsorption capacity of MgA-2 was substantially higher than that of Magnesia. These results indicated that the porous alumina support effectively enhanced the accessibility and utilization efficiency of MgO active sites.

#### 2.2.3. The Effect of Initial pH

**Figure 11:**
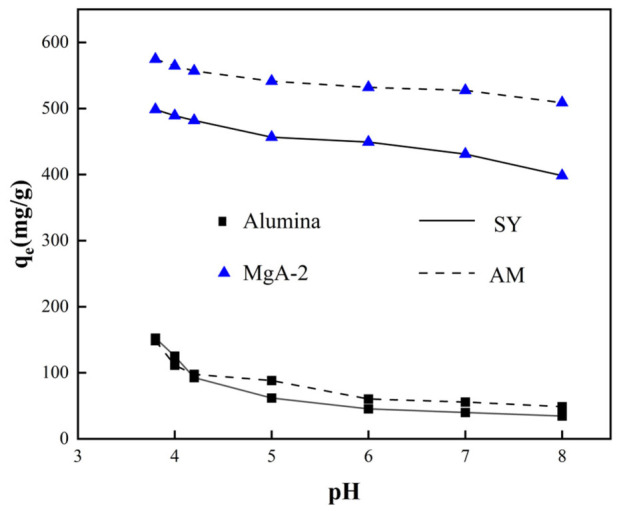
Effect of initial solution pH on SY and AM adsorption by Alumina and MgA-2.

The pH-dependent adsorption experiments and mechanistic studies were conducted using Alumina and MgA-2, where MgA-2 was selected as a representative sample with medium MgO loading. As shown in Figure 11, when the pH increased from 3.8 to 8.0, the adsorption capacities of Alumina for SY and AM decreased from 152.5 to 34.5 mg/g and from 148.7 to 48.8 mg/g, respectively, whereas those of MgA-2 decreased from 498.5 to 398.3 mg/g and from 574.6 to 508.7 mg/g, respectively. Meanwhile, the solution pH increased significantly after adsorption (from 4.0 to 6.4 and 6.5 for SY and AM adsorption on Alumina, respectively, and from 4.0 to 8.8 and 9.0 for MgA-2). These results indicated that acidic conditions favored the adsorption of both anionic dyes, since when the solution pH was below the IEP of the adsorbent, more positively charged sites were available [36]. Across the investigated pH range, MgA-2 consistently exhibited higher adsorption capacities than Alumina, confirming that MgO modification enhanced both the availability of hydroxyl sites and surface charge properties, thereby improving adsorption performance under varying pH conditions.

#### 2.2.4. Effect of Competing Anions on Adsorption

**Figure 12:**
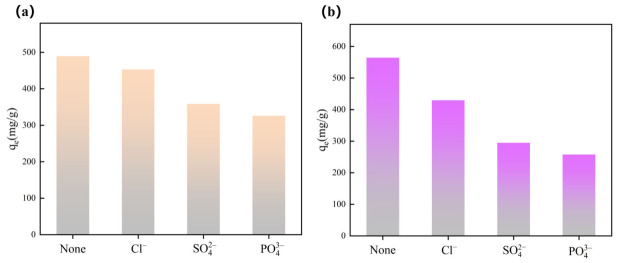
Effect of competing anions (Cl^−^, SO_4_^2−^, and PO_4_^3−^) on the adsorption on MgA-2 (**a**): SY, (**b**): AM.

To evaluate the selectivity of MgO@Al_2_O_3_ toward multi-sulfonated azo dyes, competitive adsorption experiments were conducted in the presence of Cl^−^, SO_4_^2−^, and PO_4_^3−^ (Figure 12). For both SY and AM, the capacity reduction followed the order Cl^−^ < SO_4_^2−^ < PO_4_^3−^: from 489.1 to 453.0, 358.5, and 325.9 mg/g for SY, and from 564.4 to 429.6, 304.2, and 257.7 mg/g for AM, respectively. This trend was attributed to the stronger competition of multivalent anions for surface adsorption sites. Despite partial occupation of active sites by coexisting anions, the adsorbent maintained relatively high adsorption capacities, confirming its good selectivity toward multi-sulfonated azo dyes.

#### 2.2.5. Adsorption Mechanism

To explore the adsorption mechanism, the equilibrium adsorption samples Alumina + SY (adsorbed), Alumina + AM (adsorbed), MgA-2 + SY (adsorbed), and MgA-2 + AM (adsorbed) were prepared at pH 4.0 with an adsorbent dosage of 0.1 g/L and an initial dye concentration of 200 mg/L. In parallel, SY and AM were mechanically mixed with Alumina and MgA-2 in amounts corresponding to their respective adsorption capacities to prepare the control samples, namely Alumina + SY (ground), Alumina + AM (ground), MgA-2 + SY (ground), and MgA-2 + AM (ground).

**Figure 13:**
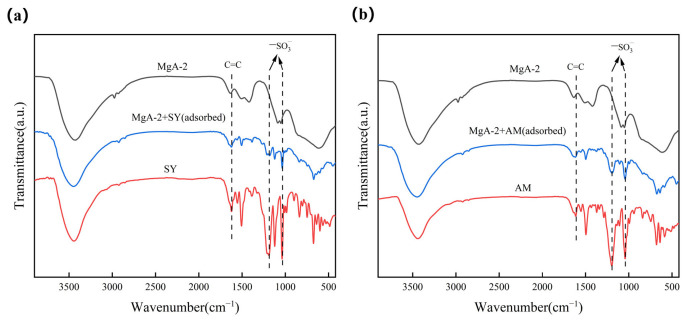
FT-IR spectra of dye, MgA-2, and MgA-2 + dye (adsorbed): (**a**) SY and (**b**) AM.

Figure 13 shows the FT-IR spectra of MgA-2, the pure dyes (SY and AM), and the corresponding dye-adsorbed samples. Compared with pristine MgA-2, new absorption bands appeared at 1625, 1190, and 1038 cm^−1^ in the SY-adsorbed sample, whereas bands at 1612, 1193, and 1043 cm^−1^ were observed in the AM-adsorbed sample. These bands were attributed to aromatic C=C stretching vibrations and sulfonate (−SO_3_^−^) vibrations, respectively. The emergence of these dye-specific bands confirmed the successful adsorption of both SY and AM onto the MgA-2 surface. It should be noted that if the dye molecules were retained on the adsorbent surface solely through physical adsorption, the Na^+^ associated with the dye molecules would also remain on the adsorbent surface and should therefore be detected in the adsorbed samples. As shown in Table 5, Na was not detected in the adsorbed samples of Alumina and MgA-2 after SY and AM adsorption, whereas it was detected in the corresponding ground samples. Meanwhile, the solution pH increased significantly after adsorption. In addition to the possible contribution of MgO to the alkalinity of the solution, this phenomenon further indicated that Na^+^ in the dye molecules was released into the solution during adsorption, while the anionic part of the dye became more strongly bound to the sample surface, thereby resulting in an increase in solution pH. These results suggested that the adsorption of SY and AM on Alumina and MgA-2 was associated with an ion exchange process involving surface sites. A similar mechanism was also reported by Chen et al. [37] using the same approach in their study on SY adsorption onto Si/P-Al_2_O_3_, where it was proposed that ion exchange occurred between surface hydroxyl groups and sulfonate groups (D–SO_3_^−^), with both sulfonate groups of SY being involved in the process.

**Figure 14:**
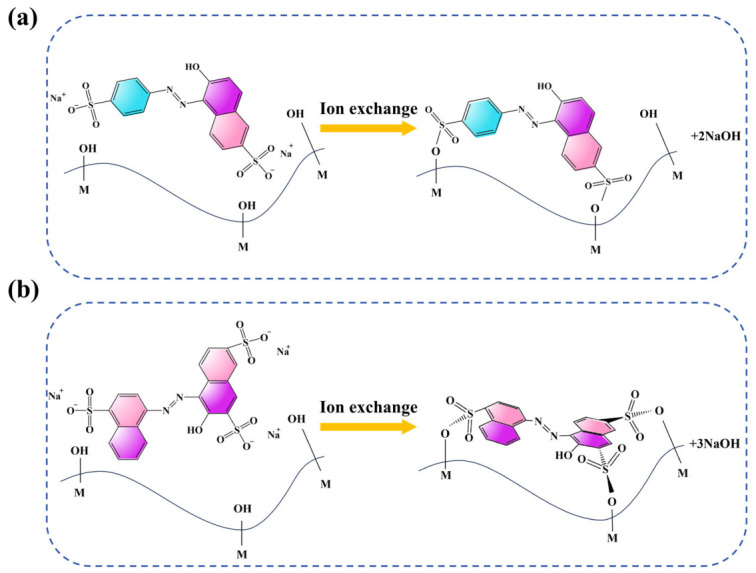
Schematic illustration of the proposed dye adsorption mechanism on the adsorbent: (**a**) SY and (**b**) AM.

Furthermore, the Langmuir maximum adsorption capacities of Alumina, MgA-1, MgA-2, and MgA-3 were converted into molar adsorption capacities after excluding Na from the dye molecular weights (Table 6). The molar adsorption capacities of SY were 1.56, 1.60, 1.46, and 1.46 times those of AM on Alumina, MgA-1, MgA-2, and MgA-3, respectively, reflecting the fact that one SY molecule contains two sulfonate groups, whereas one AM molecule contains three. Consequently, AM required three surface hydroxyl sites per molecule during adsorption, while SY required two surface hydroxyl sites. Taken together, FT-IR, the Na distribution, molar uptake ratios, and previous mechanistic evidence indicated that the adsorption of SY and AM on Alumina and MgA-x primarily proceeded via ion exchange between dye sulfonate groups (D–SO_3_^−^) and surface hydroxyl groups (M–OH, M = Al or Mg), as schematically illustrated in Figure 14. Accordingly, one SY molecule was more likely to bind through two sulfonate groups, whereas one AM molecule was more likely to involve three sulfonate groups.

#### 2.2.6. Comparison with Other Adsorbents

The adsorption capacities of the MgO@Al_2_O_3_ composites were compared with those of previously reported adsorbents for SY and AM (Table 7). For SY, MgA-3 (787.4 mg/g) exhibited a higher capacity than quaternary ammonium-modified cellulose (107.1 mg/g), PAD/QC (142.75 mg/g), PDMAEMA-grafted microspheres (312.5 mg/g), CaAl-LDH-NO_3_ (398.41 mg/g), and γ-Al_2_O_3_ (0.85 mg/g). For AM, MgA-3 (709.2 mg/g) outperformed CAS (134.9 mg/g) and MCB (404.18 mg/g), although its capacity was lower than that of ACPFP (970.9 mg/g). Although differences in pH, adsorbent dosage, and concentration range should be considered when comparing literature values, these results indicated that MgO incorporation endowed alumina with competitive adsorption capacity toward multi-sulfonated anionic dyes.

The enhanced adsorption performance could be attributed to three related effects of MgO modification: an increased density of hydroxyl-related surface groups, a more positively charged surface under acidic conditions, and the preservation of mesoporous pores that allowed dye molecules to access internal active adsorption sites. These factors worked together to strengthen ion exchange interactions, accounting for the high adsorption capacities of MgA-3 compared with those of other reported adsorbents.

## 3. Materials and Methods

### 3.1. Materials

Aluminum sulfate octadecahydrate (Al_2_(SO_4_)_3_·18H_2_O, AR), aluminum hydroxide (Al(OH)_3_, AR), and sodium hydroxide (NaOH, AR) were obtained from Xilong Scientific Company Limited (Shantou, China). Na_2_HPO_4_·12H_2_O (AR) was purchased from Beijing Chemical Works (Beijing, China). SY (87 m%) and magnesium methoxide solution (7–8 wt.% in methanol) were obtained from Shanghai Macklin Biochemical Technology Company Limited (Shanghai, China). AM (85–95 m%) was supplied by Sigma-Aldrich (Darmstadt, Germany). Commercial magnesium oxide (MgO), denoted as Magnesia, was purchased from Beijing InnoChem Science & Technology Co., Ltd. (Beijing, China).

### 3.2. Synthesis

#### 3.2.1. Preparation of Alumina

The preparation method of boehmite was generally consistent with that reported in the literature [46]. The main difference was that, prior to crystallization, a saturated Na_2_HPO_4_·12H_2_O solution was introduced into the system at a P/Al molar ratio of 0.227, where Al represented the total molar amount of all aluminum species in the system. Subsequently, the obtained boehmite sample was calcined at 600 °C for 2 h to obtain phosphorus modified Al_2_O_3_, which was denoted as Alumina.

#### 3.2.2. Preparation of MgA-x

Using the conventional impregnation method, Alumina was placed in a beaker and continuously stirred at room temperature while an appropriate amount of magnesium methoxide solution was added dropwise. The suspension was dried at 35 °C for 6 h and subsequently calcined at 400 °C for 2 h to obtain a series of MgO-supported porous alumina composites (MgO@Al_2_O_3_), which were denoted as MgA-x. The amount of magnesium methoxide added in each impregnation step was calculated based on MgO/Al_2_O_3_ mass ratios of 0.24, 0.48, and 0.72, and the composites were named MgA-1, MgA-2, and MgA-3, respectively.

### 3.3. Analytical Procedures

The phase composition of the samples was analyzed by X-Ray diffraction (XRD) using a SmartLab 9 kW diffractometer (Rigaku, Tokyo, Japan) operated at 45 kV and 200 mA with Cu-Kα radiation. The diffraction patterns were collected in the 2θ range of 20–70° at a scanning rate of 5°/min. The specific surface area and pore structure characteristics were determined using N_2_ adsorption–desorption at 77 K on a TriStar II 3020 analyzer (Micromeritics, Norcross, GA, USA), and the specific surface area was calculated by the BET method. The microstructure was characterized using a JEM-2100 high-resolution transmission electron microscope (JEOL, Tokyo, Japan). Fourier-transform infrared (FTIR) spectra were recorded using a Nicolet 6700 spectrophotometer (Thermo Fisher Scientific, Waltham, MA, USA). Samples were prepared using the KBr pellet method: the sample was thoroughly ground and mixed with KBr at a mass ratio of 1:399, and 0.15 g of the mixture was pressed into a transparent pellet. The obtained spectra were used for the semi-quantitative analysis of hydroxyl groups in the samples. The zeta potential was measured using a ZEN3600 Malvern instrument (Malvern Panalytical, Malvern, UK) after dispersing the samples in 0.01 M KNO_3_ solution, followed by pH adjustment with 0.01 M HNO_3_ and 0.01 M KOH. The dye concentration in solution was determined by a UV–Vis spectrophotometer (UV-2600, Shimadzu, Kyoto, Japan) based on the absorbance at the characteristic wavelength.

### 3.4. Adsorption Studies

#### 3.4.1. Effect of Initial Solution pH

Batch adsorption experiments were carried out at room temperature with a stirring speed of 400 r/min and an adsorbent dosage of 0.1 g/L.

To investigate the effect of the initial solution pH on adsorption capacity, 10 mg of adsorbent was added to 100 mL of SY or AM solution with an initial dye concentration of 200 mg/L. The solution pH was adjusted to 3.8, 4.0, 4.2, 5.0, 6.0, 7.0, and 8.0 using 0.1 mol/L HCl or NaOH. After adsorption, the solution was filtered, and the residual dye concentration was determined using a UV–Vis spectrophotometer (UV-2600, Shimadzu, Japan). The equilibrium adsorption capacity (q_e_, mg/g) was calculated according to Equation (1):

(1) $$q_{e}=\frac{\left(C_{0}-C_{e}\right)\times V}{m}$$

where C_0_ (mg/L) is the initial dye concentration, C_e_ (mg/L) is the equilibrium dye concentration, V (L) is the solution volume, and m (g) is the adsorbent mass.

#### 3.4.2. Effect of Competing Anions on Adsorption

To evaluate the adsorption selectivity of the adsorbent toward SY and AM, competitive adsorption experiments were conducted in the presence of common inorganic anions. Cl^−^, SO_4_^2−^, and PO_4_^3−^ were introduced into 200 mg/L dye solutions at a concentration of 100 mg/L, and the solution pH was adjusted to 4.0 before adsorption.

#### 3.4.3. Adsorption Kinetics Experiments

The concentration of SY or AM solution was fixed at 200 mg/L and the pH was adjusted to 4.0. The mixture was continuously stirred using a magnetic stirrer, and samples were withdrawn at predetermined time intervals (5, 10, 15, 30, 45, 60, 180, 360, 720, and 900 min) and filtered through a membrane prior to analysis.

The experimental data were fitted using pseudo-first-order and pseudo-second-order kinetic models. The corresponding equations employed for fitting are presented as follows (Equations (2) and (3)):

(2) $$\mathrm{lg}\left(q_{e,exp}-q_{t}\right)=lgq_{e}-\frac{k_{1}}{2.303}t$$

(3) $$\frac{t}{q_{t}}=\frac{1}{k_{2}{q_{e}}^{2}}+\frac{1}{q_{e}}t$$

where k_1_ (min^−1^) is the rate constant of adsorption in the pseudo-first-order model, and k_2_ (g/mg∙min) is the rate constant of adsorption in the pseudo-second-order model.

#### 3.4.4. Equilibrium Adsorption Experiments

The effect of initial dye concentration on adsorption capacity was investigated at pH 4.0. For MgA-x, the initial concentrations of SY were set at 200, 300, 400, 500, and 600 mg/L, while those of AM were 50, 100, 200, 300, and 400 mg/L. In addition, the initial concentrations of both SY and AM on Alumina were 20, 50, 80, 100, and 200 mg/L.

The experimental data were fitted using Langmuir and Freundlich isotherm models, which are expressed by Equations (4) and (5), respectively.

(4) $$\frac{C_{e}}{q_{e}}=\frac{1}{{K_{L}q}_{m}}+\frac{C_{e}}{q_{m}}$$

(5) $$lgq_{e}=lgK_{F}+\frac{1}{n}{lgC}_{e}$$

where *q*_m_ (mg/g) is the theoretically predicted amount of adsorbed dye, *K_L_* is Langmuir constant, and *K*_F_ is Freundlich constant.

## 4. Conclusions

MgO-modified alumina composites were prepared by impregnation followed by calcination and evaluated for the adsorption of two multi-sulfonated anionic azo dyes. MgO incorporation increased the isoelectric point, enhanced hydroxyl-related surface features, and preserved a mesoporous framework, although the BET surface area and pore volume decreased with increasing MgO loading. The best-performing sample, MgA-3, achieved maximum Langmuir adsorption capacities of 787.4 mg/g for SY and 709.2 mg/g for AM at pH 4.0. Kinetic and isotherm analyses indicated that the adsorption process was better described by the pseudo-second-order and Langmuir models, respectively, suggesting that the adsorption process was predominantly governed by surface active sites with monolayer adsorption characteristics. Mechanistic studies revealed that adsorption mainly occurred via ion exchange between surface hydroxyl groups (M–OH, M = Al or Mg) and dye sulfonate groups (D–SO_3_^−^). The lower molar adsorption capacity of AM relative to SY was consistent with its higher number of sulfonate groups and greater demand for surface hydroxyl sites. Compared with previous reported adsorbents, MgA-3 exhibited competitive adsorption capacities for both dyes. Notably, MgA-2 exhibited the highest MgO-normalized adsorption capacity, indicating that the utilization efficiency of MgO active sites was the highest at this loading level. These findings highlighted MgO modification as an effective strategy for tuning the surface charge and hydroxyl group density of alumina adsorbents for the removal of multi-sulfonated anionic dyes.

## Figures and Tables

**Table 1:** BET surface area and pore volume of samples.

Sample	BET Surface Area (m^2^/g)	BET Pore Volume (cm^3^/g)
Alumina	548	2.97
MgA-1	381	2.12
MgA-2	309	1.50
MgA-3	254	1.11

**Table 2:** Pseudo-first-order and pseudo-second-order kinetic fitting data of the adsorption process of SY and AM by samples.

Model	Parameter	SY				AM			
		Alumina	MgA-1	MgA-2	MgA-3	Alumina	MgA-1	MgA-2	MgA-3
Pseudo-first- order	q_e,exp_	125.8	198.0	489.4	620.2	112.0	218.6	564.6	676.1
	q_e,cal_	42.8	82.0	100.8	142.0	43.1	72.3	99.2	128.4
	k_1_	5.50 × 10^−3^	6.80 × 10^−3^	8.00 × 10^−3^	1.80 × 10^−3^	3.10 × 10^−3^	3.70 × 10^−3^	6.70 × 10^−3^	7.00 × 10^−3^
	R^2^	0.8947	0.9594	0.8493	0.7998	0.9212	0.8263	0.8690	0.8383
Pseudo-second-order	q_e,cal_	126.7	200.4	492.6	625.0	111.5	218.8	565.0	680.3
	k_2_	6.24 × 10^−4^	3.65 × 10^−4^	4.31 × 10^−4^	4.48 × 10^−4^	5.28 × 10^−4^	3.52 × 10^−4^	4.01 × 10^−4^	3.10 × 10^−4^
	R^2^	0.9989	0.9996	0.9999	1.0000	0.9975	0.9994	0.9999	0.9999

**Table 3:** Langmuir and Freundlich fitting parameters for SY adsorption.

Model	Parameter	SY				
		Alumina	MgA-1	MgA-2	MgA-3	Magnesia
Langmuir	q_m_	134.4	289.9	699.3	787.4	122.4
	K_L_	6.83 × 10^−2^	1.12 × 10^−2^	1.48 × 10^−2^	2.74 × 10^−2^	6.94 × 10^−2^
	R^2^	0.9947	0.9973	0.9977	0.9998	0.9997
Freundlich	K_F_	48.33	64.20	183.51	330.56	39.35
	1/n	1.85 × 10^−1^	2.16 × 10^−1^	1.96 × 10^−1^	1.30 × 10^−1^	2.42 × 10^−1^
	R^2^	0.9695	0.9723	0.9488	0.9363	0.9248

**Table 4:** Langmuir and Freundlich fitting parameters for AM adsorption.

Model	Parameter	AM				
		Alumina	MgA-1	MgA-2	MgA-3	Magnesia
Langmuir	q_m_	113.6	238.7	628.9	709.2	101.3
	K_L_	5.53 × 10^−1^	6.02 × 10^−2^	1.15 × 10^−1^	2.70 × 10^−1^	9.81 × 10^−2^
	R^2^	0.9995	0.9994	0.9981	0.9997	0.9999
Freundlich	K_F_	72.52	109.88	343.49	434.28	41.21
	1/n	9.51 × 10^−2^	1.27 × 10^−1^	1.03 × 10^−1^	8.85 × 10^−2^	1.70 × 10^−1^
	R^2^	0.6796	0.9748	0.9474	0.9070	0.9119

**Table 5:** EDS analysis of Na contents in ground and adsorbed samples of Alumina and MgA-2.

Sample	Na (Weight%)	Na (Atomic%)
Alumina + SY (ground)	2.31	1.79
Alumina + SY (adsorbed)	0.00	0.00
Alumina + AM (ground)	1.62	1.17
Alumina + AM (adsorbed)	0.00	0.00
MgA-2 + SY (ground)	4.83	3.62
MgA-2 + SY (adsorbed)	0.00	0.00
MgA-2 + AM (ground)	4.12	3.02
MgA-2 + AM (adsorbed)	0.00	0.00

**Table 6:** Molar adsorption capacities of SY and AM after Na exclusion and the corresponding SY/AM ratios.

Sample	Adsorption Capacity of SY (mmol/g)	Adsorption Capacity of AM (mmol/g)	Molar Ratios of SY/AM
Alumina	0.3308	0.2122	1.56
MgA-1	0.7133	0.4457	1.60
MgA-2	1.721	1.175	1.46
MgA-3	1.937	1.324	1.46

**Table 7:** Comparison of SY and AM adsorption capacity by different adsorbents.

Dye	Adsorbent	Adsorption Capacity mg/g	Initial pH	References
SY	Quaternary ammonium-modified cellulose	107.08	6	[38]
SY	PAD/QC	142.75	-	[39]
SY	CaAl-LDH-NO_3_	398.41	4	[40]
SY	γ-Al_2_O_3_	0.85	2	[35]
SY	PDMAEMA-grafted microspheres	312.5	2.0	[30]
SY	OMC-2Nd	285.7	6.5	[41]
SY	MgA-3	787.4	4.0	This study
AM	CAS	134.9	2	[42]
AM	MCB	404.18	3	[43]
AM	ACPFP	970.87	5	[44]
AM	Fe_3_O_4_@nSiO_2_@mSiO_2_@DHIM-NH_2_	84.40	2	[45]
AM	MgA-3	709.2	4.0	This study

## Data Availability

The original contributions presented in this study are included in the article. Further inquiries can be directed to the corresponding author.
